# Ultrasound evaluation of radial nerve injuries by cortex overlapping screw tips after internal fixation of humeral fractures: a cadaveric study

**DOI:** 10.1007/s00590-024-04057-8

**Published:** 2024-08-21

**Authors:** David Lorenzana, Anna Spicher, Frank J. P. Beeres, Bernhard Moriggl, Hagen Bomberg, Urs Eichenberger

**Affiliations:** 1 Pain Therapy, Department of Anaesthesiology, Kantonsspital Lucerne, Lucerne, Switzerland; 2 Division of Anaesthesia, Balgrist University Hospital, Forchstrasse 340, 8008 Zurich, Switzerland; 3 Department of Traumatology, Medical University of Innsbruck (MUI), Innsbruck, Austria; 4 Department of Orthopaedic Surgery and Traumatology, Kantonsspital Lucerne, Lucerne, Switzerland; 5 Division of Clinical and Functional Anatomy, Medical University of Innsbruck (MUI), Innsbruck, Austria

**Keywords:** Neuropathic pain, Diagnostic nerve ultrasound, Peripheral nerve injuries, Humeral fractures, Radial nerve

## Abstract

**Purpose:**

The radial nerve may be painfully irritated or damaged by open reduction and internal fixation (ORIF) of humeral fractures. Secondary radial nerve lesions after ORIF of humeral shaft fractures are described in up to 16%. Not only peripheral nerves but also orthopaedic instruments and osteosynthesis material are well visible by ultrasound. The aim of this study was to evaluate the accuracy of ultrasound in assessing the relation between the bone overlapping screw tips and the radial nerve close to the humeral bone.

**Methods:**

Ultrasound-guided drilling was used to place screws as close as possible to the radial nerve in 8 humeral bones of four cadavers. The relation between the radial nerve and the screw tips was assessed by high-resolution ultrasound, and the overlap of all screw tips over the bone was measured by ultrasound and fluoroscopy. Thereafter, the findings were validated by anatomical dissection.

**Results:**

We could correctly identify all screw tips and their relation to the radial nerve by ultrasound. In 7 of 8 cases, the screw tip had direct contact with the radial nerve. The overlaying length of the screw tip was accurately measured by using ultrasound in all cases. In contrast fluoroscopy underestimated this length in 50% of cases.

**Conclusion:**

With this study, we show that ultrasound can reliable visualize the screw tips and its relation to the radial nerve. Ultrasound is a promising diagnostic tool to evaluate patients with radial nerve irritations or lesions after ORIF of humeral fractures. Furthermore, ultrasound could be an adequate tool to guide drilling.

## Introduction

Nerve damage after humeral shaft fractures is a serious complication. The radial nerve is paralysed in up to 11.8% of patients [15]. This is the most common peripheral nerve injury associated with long bone fractures [15]. Secondary lesions of the radial nerve after open reduction and internal fixation of humeral shaft fractures have been described in up to 12–16% of cases [4]. Reasons for damage to the radial nerve can be a screw tip [5] or stretching of the radial nerve due to the protruding position of a plate [12]. Magnetic resonance imaging appears to be an accurate tool for assessing nerve lesions. However, the osteosynthesis material can severely impair the images and nerve lesions cannot be recognized [10]. Ultrasound can therefore be an alternative. Ultrasound is a proven method for visualizing the radial nerve [2, 3]. Ultrasound visibility of the screw tips and their impairment of tendons in open reduction and internal fixation of distal radius fractures are well described [1, 17, 18], including images with osteosynthesis material [5, 6]. In a pilot study the feasibility of ultrasound evaluation of radial nerve continuity early after humeral shaft fracture fixation was shown [9].

We hypothesize that ultrasound is an accurate diagnostic tool to assess the relationship between screw tips and the radial nerve after open reduction and internal fixation of humeral fractures. However, no systematic study is available. Therefore, the primary objective of this study was to evaluate the accuracy of ultrasonography in assessing the relationship between the drill, screw tip, and radial nerve during and after open reduction and internal fixation of humeral fractures in a human cadaver model. The secondary objectives of this study were to evaluate the ultrasound visibility of the drill hole, to compare ultrasound and fluoroscopy in measuring the cortical bone overlapping the screw tip portion, and to evaluate whether precise guidance of the drilling direction is possible using ultrasound.

## Materials and methods

The bodies were donated to the Division of Clinical and Functional Anatomy at the Medical University of the place of study by people who had given their written informed consent prior to death for scientific and educational purposes [14]. Institutional approval for the usage of cadavers that were in the legal custody of the Department of Anatomy, Histology and Embryology of the of the corresponding Medical University, was obtained. Ethical approval was not necessary according to Austrian law.

All cadavers were preserved using an arterial injection of an ethanol-glycerol solution followed by 1–3 months of immersion in diluted phenolic acid [7, 13].

We conducted the specified procedures bilaterally on both upper arms of four cadavers (three female and one male, aged 88–97, Body Mass Index 20–26).

The cadavers were placed in supine position with the arms in 90° abduction and external rotation. The hand was fixed on a second table in order to have the whole length and circumference of the upper arm freely accessible for manipulation as well as for ultrasound scanning (from dorsal). Moreover, the drilling and placement of the screws were possible without repositioning of the arm.

### Ultrasound

Ultrasound scanning was performed by two experienced sonographers using a high-frequency 13–8 MHz linear array probe (SL1543 probe, Esaote MyLab Seven US System; Esaote, Genoa, Italy).

### Dissection

Dissection was performed by an anatomist blinded to the screw placement, the fluoroscopy and ultrasound evaluation.

As first step of the preparation, an extended incision of the skin and subcutis along the length of the dorso-lateral upper arm (from proximal dorsal to distal lateral, along the suspected course of the radial nerve) was performed up to the surface of the muscles (mainly the triceps muscle). The radial nerve was approached from two sides: distally by gentle, blunt separation (with both thumbs) of the brachial and brachioradialis muscle and very proximal by cutting both, the long and lateral heads of triceps brachii muscle. Finally, the radial nerve was exposed along the entire spiral groove. Care was taken not to displace the nerve in order to preserve its exact position in relation to the screw tip (for documentation).

### Primary objectives

Ultrasound scanning was performed to identify the radial nerve and the screw tip in the spiral groove and their relationship. After anatomical dissection, the relation of the screw to the radial nerve was documented and compared with the ultrasound findings.

### Secondary objectives

In order to assess the sonographic visibility of the drill holes, after drilling and before inserting the screws the hole in the second cortical bone was sonographically searched and the size was measured in the ultrasound image. These measurements were compared with the diameter of the drill used (Table 1).

**Table 1:** Ultrasound evaluation of the drill hole (mm)

Ultrasound evaluation of the drill hole		
Drill hole	Ultrasound drill hole (mm)	Diameter of the drill (mm)
1	3.2	3.2
2	3.2	3.2
3	**3.5**	**2.5**
4	2.3	2.5
5	2.5	2.5
6	2.3	2.5
7	3.3	3.2
8	3.5	3.2

Bold values with relevant deviation between mesurement in the ultrasound image and diameter of the drill used

**Fig. 1:**
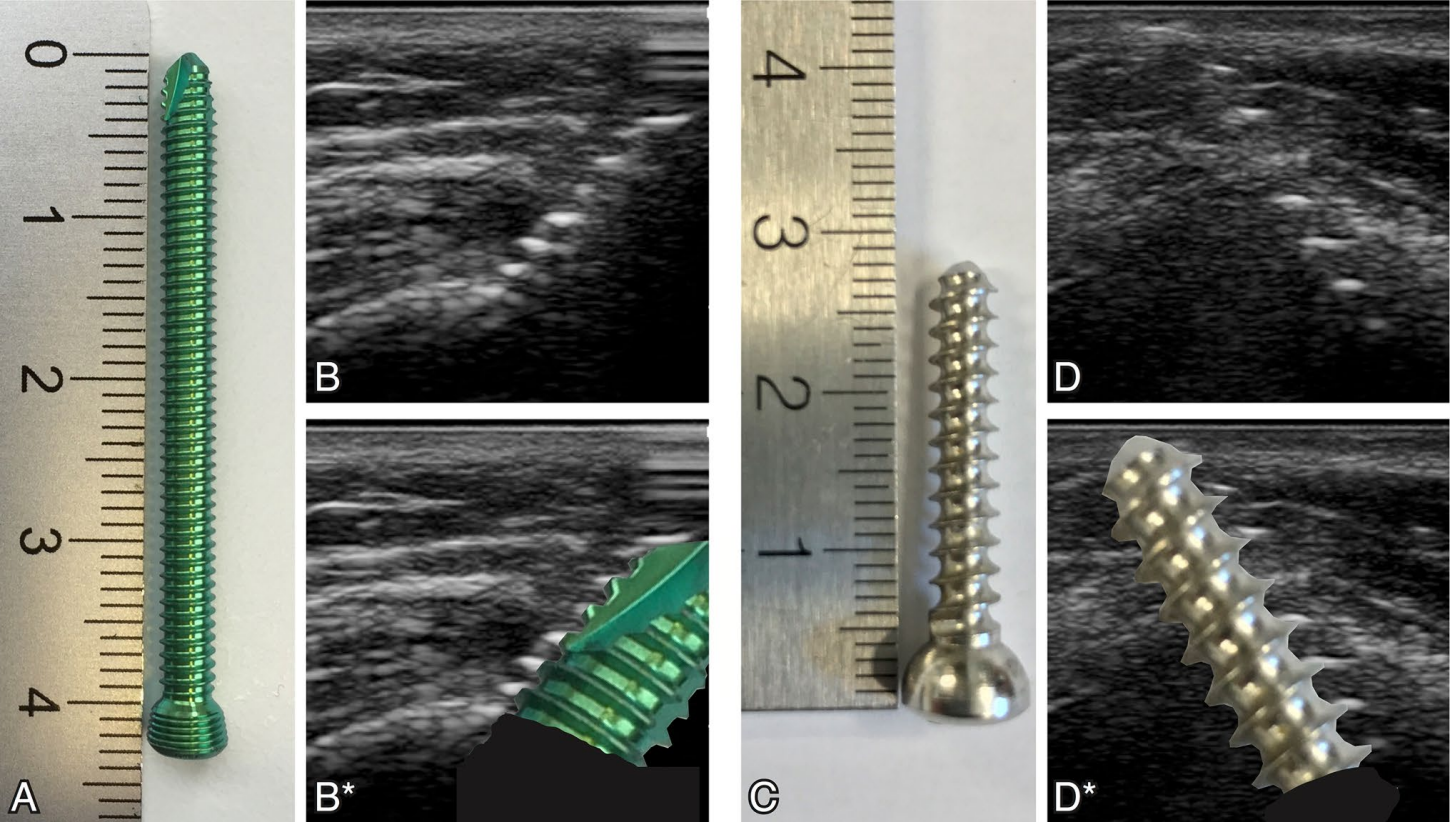
Types of screws used in the study. **A**: Self-tapping (thread cutting) screw of 3.5 mm diameter. **B**: Ultrasound-image of the self-tapping screw, B* same image with the photo of the screw tip overlapped. **C**: Regular (non-cutting) screw of 4.5 mm diameter. **D**: Ultrasound-image of the regular screw, D* same image with the photo of the screw tip overlapped. The reflections on the screw threads are clearly visible in the ultrasound image (**B** and **D**) as regularly arranged bright hyperechogenic lines

To compare ultrasound and fluoroscopy in measuring the cortical bone overlapping screw tip portion, the following steps were performed: After determining the required length of the screw based on the depth measurement of the deeper surface of the cortical bone, the surgeon inserted the screw manually as necessary. Two screw types used in daily surgical practice for humeral fractures were used: self-tapping (thread cutting) and non-self-tapping (non-cutting) screws of a diameter of 3.5 mm or 4.5 mm, respectively. The screw length was chosen to overlap the second cortex between 2 and 6 mm. This enabled us to have different lengths of visible screw tips investigated in the study. The final positions of the screws were documented by fluoroscopy. The number of overlapping threads through the second cortex was counted using an exact vertical fluoroscopic image. Thereafter, the overlapping screw tip was localized by ultrasound and the number of threads overlapping the second cortex was counted and the length of the overlapping tip was measured in mm in the ultrasound image. The screw threads are clearly visible in the ultrasound image based on the ultrasound reflections (Fig. 1). The values from the fluoroscopy and ultrasound measurements were compared with the findings obtained by dissection (Table 2).

**Table 2:** Comparison of values of the second cortex overlapping part of the screw tips

A			
	Fluoroscopy	Ultrasound	Dissection
Screw	Number of threads	Number of threads	Number of threads
1 (a)	**3**	3.5	3.5
2 (a)	1.5	1.5	1.5
3 (b)	3.5	3.5	3.5
4 (b)	1	1	1
5 (b)	1.5	1.5	1.5
6 (b)	**5.5**	6	6
7 (a)	**6**	9	9
8 (a)	**2**	3	3

B		
	Ultrasound	Dissection
Screw	Length of the screw tip (mm)	Length of the screw tip (mm)
1 (a)	6	5.9
2 (a)	3.2	3.7
3 (b)	5.6	5.6
4 (b)	3.4	2.8
5 (b)	3.1	3.5
6 (b)	6.9	7
7 (a)	**11.5**	**13.6**
8 (a)	4.5	4.5

Bold values with relevant deviation compared to the findings by dissection

*A* number of threads overlapping the second cortex (fluoroscopy vs ultrasound vs dissection). *B* measurements of the overlapping length of the screw tips in mm. (ultrasound vs dissection)

*a* self-tapping (thread cutting) screw of 3.5 mm, *b* regular (non-cutting) screw of 4.5 mm

To assess whether precise guidance of the drilling direction by ultrasound is possible position and direction of the drill was assigned to the surgeon involved in the study in direction to the radial nerve. After an incision the first cortical bone was drilled, and if needed, the course of the drill was readjusted in direction to the nerve. After ensuring the proper direction by ultrasound, the second cortical bone was drilled. This ultrasound-guided drilling was used to place the screws as close as possible to the radial nerve.

### Data analysis

Continuous variables are expressed as means ± standard deviations. Categorical variables are presented as absolute and relative frequencies. The main data presentation includes descriptive statistics. Descriptive statistics were used to describe the cases in which the tip of the screw was in direct contact with the radial nerve. Moreover, continuous variables were compared using Mann–Whitney U-test. Categorical variables were compared with chi2 tests. Two-sided *p* values < 0.05 were considered statistically significant. Data analysis was performed using SPSS Statistics 29™ (SPSS Inc, Chicago, Illinois, USA).

## Results

### Primary outcomes

**Fig. 2:**
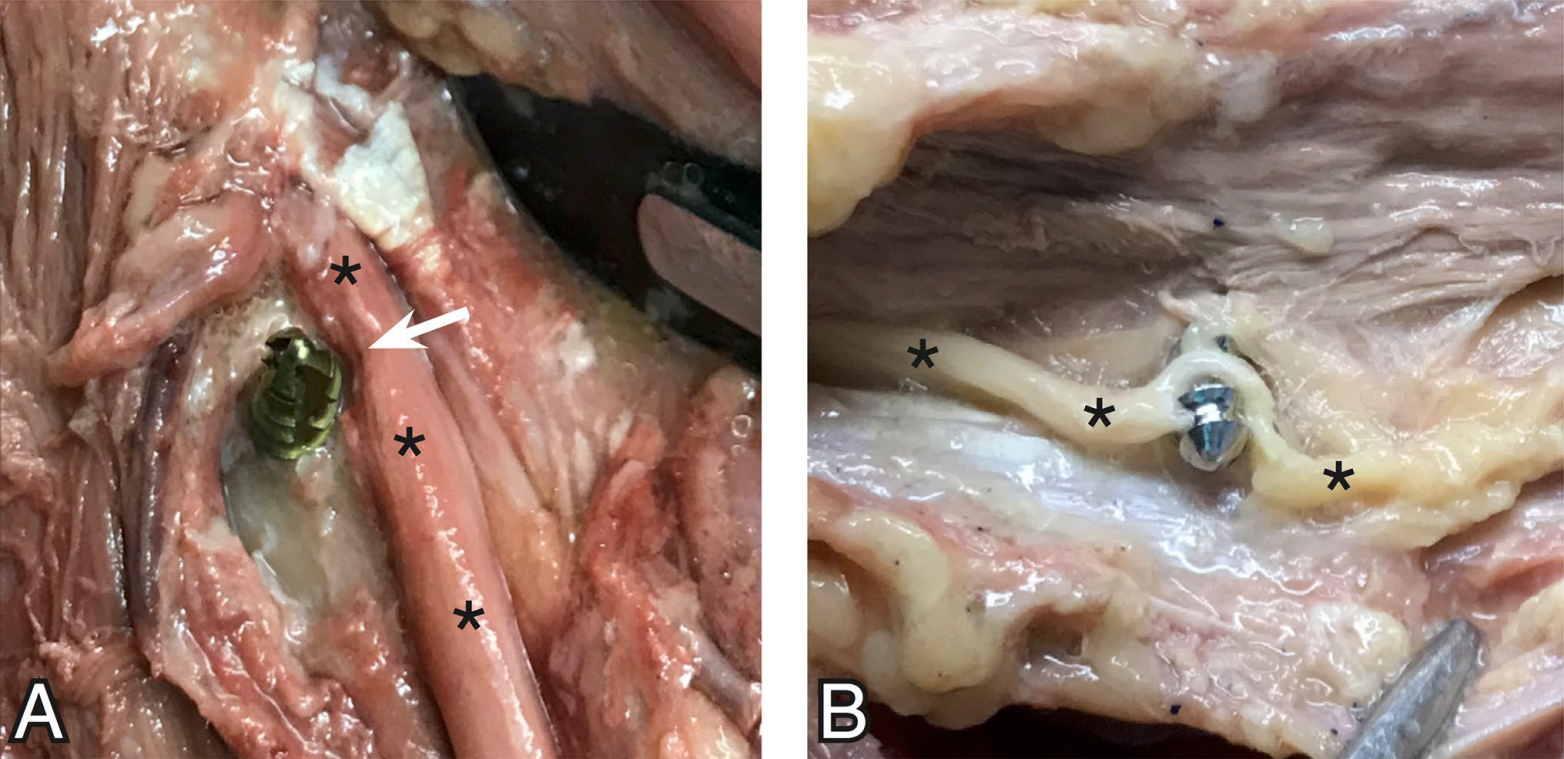
The two cases with macroscopic visible nerve-lesions identified by dissection. Radial nerve marked with asterisk (*). **A**: Radial nerve with notch (white arrow) at the location where the self-tapping screw has contact to the nerve (the nerve has slightly been displaced from the screw tip for better visibility of the notch). **B**: Radial nerve pierced by the regular (non-cutting) screw. Some nerve fibres have been severed (color figure online)

In all cases, the radial nerve was easily visualized in the spiral groove. All the screw tips have been correctly identified in ultrasound with accurate evaluation of their relation to the radial nerve. In 7 out of 8, the screw tips had direct contact with the radial nerve. In the remaining case, a small tissue layer of 1 mm stayed between the screw and the nerve. In two cases, macroscopic lesion of the radial nerve could be identified by dissection (Fig. 2).

### Secondary outcomes

The drill hole as interruption of the continuity of the bony surface was visible by ultrasound in all cases and could be accurately measured in 7 of 8 cases with a deviation of maximal 0.3 mm (Table 1). In one case, the size of the drill hole was overestimated by ultrasound (3.5 instead of 2.5 mm; Table 1).

In 4 of 8 cases (50%) of all screws, the overlap of the screws through the second cortex was underestimated by fluoroscopy from 0.5 to 3.0 threads compared with anatomical dissection as the gold standard (*p* = 0.02). In contrast, ultrasound was able to identify accurately the overlapping length of the screw tip in all cases compared with anatomical dissection (*p* = 1; Table 2).

Comparing the ultrasound measurements with dissection, the count of the numbers of threads overlapping the second cortex was equal (*p* = 1) and the measurement of the overlapping length comparable (ultrasound: 5.5 ± 2.8 mm versus dissection: 5.8 ± 3.4 mm; *p* = 0.9; Table 2). However, in only one screw the angle of the screw in relation to the surface of the humeral bone was far away from rectangular and therefore the measurements from one side of the screw to the other were highly different (11–16.2 mm) and the mean value differed by 2.1 mm from the ultrasound measurement (11.5 mm, Table 2B) take at a single position.

The guidance of the drill exactly in direction of the nerve was possible in 7 of 8 cases.

## Discussion

This anatomical study shows that ultrasound has a high potential to evaluate the relation of the radial nerve and the screw tips after open reduction and internal fixation of a humeral fracture. Moreover, ultrasound has the potential to play a role in the prevention of radial nerve damage when used as a guiding tool during drilling and screw insertion.

The prevention of radial nerve damage in the spiral groove during open reduction and internal fixation of humeral fractures is essential. Nevertheless, damage does occur [4] and is not only dependent on the applied surgical technique, but may also be caused by a not expected course of the radial nerve due to interindividual variable anatomy. Ultrasound allows an easy, cheap, real-time and bedside visualization of the individual course of nerves [2].

**Fig. 3:**
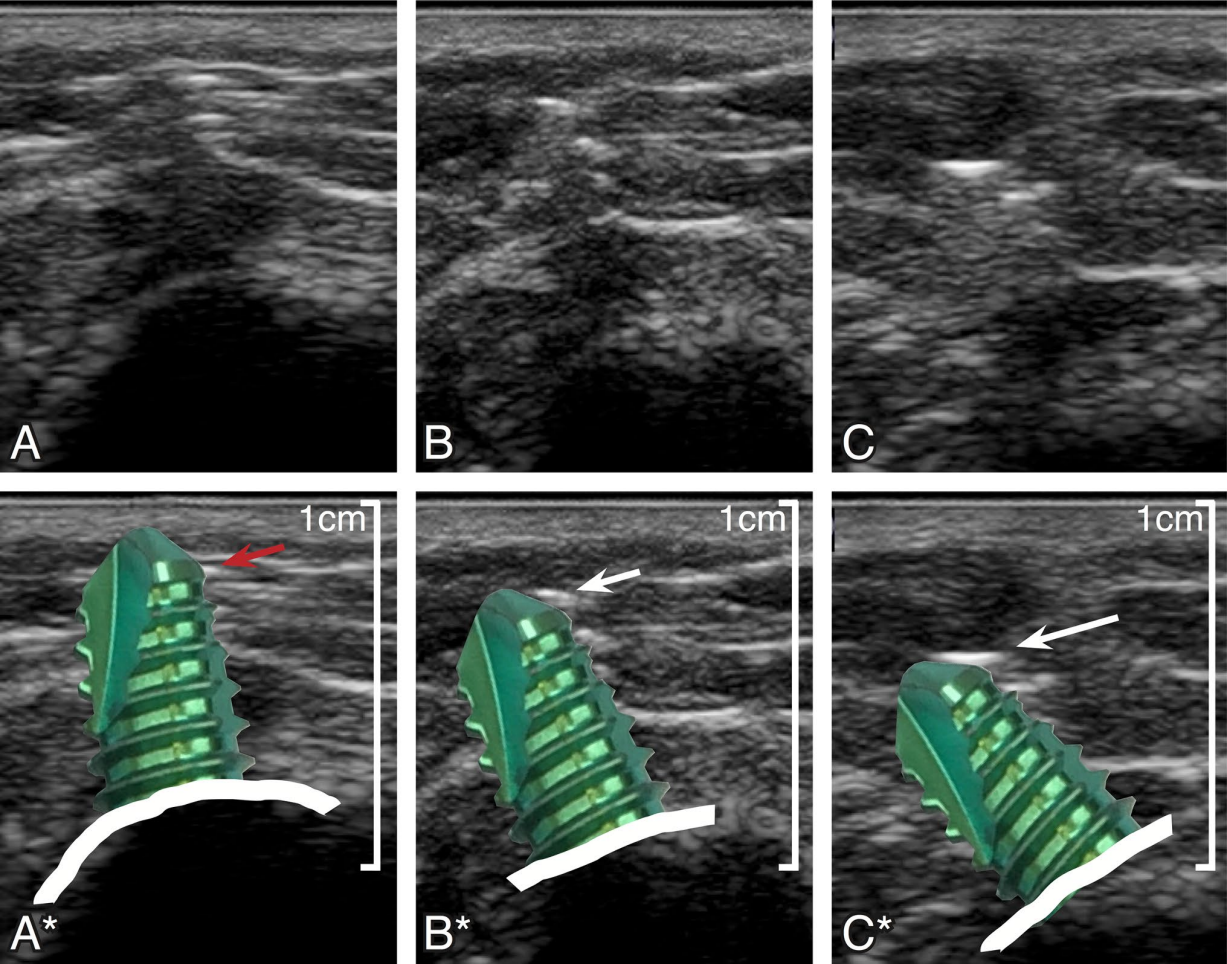
Different reflection patterns of the screw tip dependent on the angle of the ultrasound beam. Lateral tilting of the ultrasound beam (B/B* and C/C*) helps to better visualize the most superficial end of the screw tip. To obtain an oblique scan of the screw may help to improve visibility. The better the cone surface of the screw tip is rectangular orientated to the ultrasound beam, the brighter the reflection (white arrow) and the better the visibility of the real tip of the screw will be. By scanning more or less in the direction of the screw (**A** and A*) there might be only little reflection on the rounded tip of the screw. It is more likely to get the first bright reflection on the first thread (red arrow) than the tip of the screw (color figure online)

The good ultrasound visibility of screws is mainly caused by the reflection of the ultrasound waves on the screw threads. The typical ultrasound image has aspects of the steps of a stair as shown in Fig. 1. By scanning parallel to the screw direction, it is important to notice that the highest bright reflection more likely represents the reflection on the first thread than the real tip of the screw. The real tip of the screw often appears—due to the curved surface—only as a very small punctual white reflection. Lateral tilting of the ultrasound beam helps to get a better image of the screw tip. As shown in Fig. 3, the bright reflection on the cone surface of the screw tip is caused by its orientation in direction to the ultrasound beam. Tilting of the ultrasound beam is important and helps to get a reliable identification of the real tip of the screw, which is required for an accurate evaluation of the nerve—screw tip relation.

**Fig. 4:**
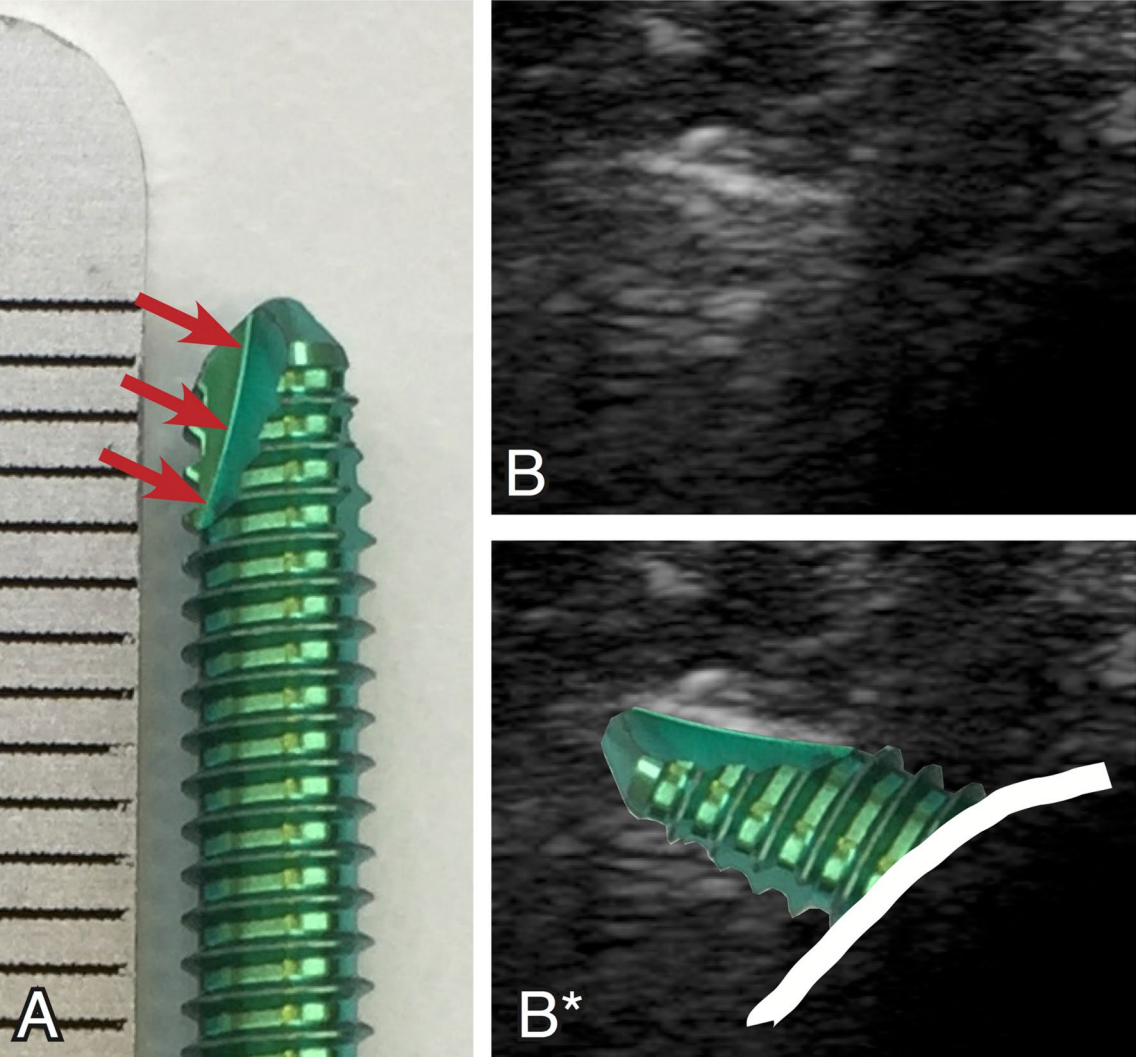
Additional ultrasound aspect of self-tapping screws. **A**: Self-tapping screw with a gap (red arrows) in the screw tip, which creates the cutting edge of the self-tapping screw. **B**: Orientation of the ultrasound plane almost rectangular to the gap in the screw tip causes absence of the characteristic thread-reflections. B* same image with the photo of the screw superimposed (color figure online)

Both screw types used in the study (cutting and non-cutting screws) have comparable reflection aspects, distinguishing in the distance between the threads and the corresponding reflections in the ultrasound image (Fig. 1). Self-tapping screws have a gap in the continuity of the threads on the tip of the screw, generating a cutting edge. If this gap is orientated in direction of the ultrasound probe, the reflection on the threads are missed. A bright linear reflection might be visible but only in nearly lateral scanning of the screw tip (Fig. 4).

All drill holes could be visualized as gap in the continuity of the bony surface. In 7 out of 8 ultrasound measurements of the gaps corresponded accurately to the real drill diameter. The one case with a deviation of 1 mm might be due to little bone pieces broken out during the drilling or due to ultrasound artefacts.

If in the diagnostic setting after open reduction and internal fixation an empty drill hole (without screw in it) is identified, damage of the nerve running over the hole could have occurred due to the drilling process and has to be considered. As drill-holes are small, an identification of all the empty holes along a fractured and osteosynthesized bone might be difficult and not always possible.

It is known that high-resolution ultrasound is a useful tool in the evaluation of traumatic nerve lesions [11, 12, 19, 20]. There is no literature about ultrasound evaluation of neuropathic pain after open reduction and internal fixation of humeral fractures.

In two of our study cases, a macroscopic nerve lesion was documented by dissection. We cannot distinguish, if the lesion was due to the drill or the screw tip or both. Other signs of nerve lesion as epineural or intraneural hematoma formation, nerve stretching and swelling, neuroma formation as well as nerve entrapments secondary to scare formation need time to develop and are visible by ultrasound only in living subjects and not in cadavers. Every nerve with contact to osteosynthesis material must be considered at risk for damage or painful nerve irritation. Nerves overlaying screw tips without direct contact or nerves overlaying empty drill holes should be considered at possible risk due to deeper drilling, probing by transfixion wires, temporary screw insertion or an affection due to the depth gauge. Combining these findings with the knowledge of ultrasound pattern of traumatic nerve lesions should be the way of extended ultrasound evaluation of neuropathic pain and/or nerve damage after open reduction and internal fixation.

Fluoroscopy is the gold standard to control and document the screw position in relation to the bone during and after open reduction and internal fixation. The overlaying part of the screw tip is often underestimated in the fluoroscopic image [16] due to a deviation of the exact vertical beam path or a drilling outside the largest diameter of the bone. This could be confirmed with this study. In contrast to the accuracy of ultrasound in counting the number of screw threads visible through the second cortex in all our cases, fluoroscopy underestimated this value in half of the cases compared to the gold standard—direct counting after dissection (Table 2). The length of screw through the bone measured by ultrasound was very close to the valued measured after dissection in all but one case. In this case, the angle of the screw in relation to the surface of the humeral bone was far away from rectangular and therefore the measurements from one side of the screw to the other were highly different.

From our view, ultrasound will not replace fluoroscopy-guidance in open reduction and internal fixation of humeral fracture, but it may be an additional tool to use to evaluate complex cases after surgery.

Precise ultrasound guidance of the drill in direction of the radial nerve was possible in our study (7 screws out of 8 had direct contact to the radial nerve, the remaining had only 1 mm distance to it). We suppose that ultrasound guidance of the drill to achieve the goal not to hit the nerve should be even easier.

Ultrasound can be used intraoperative under sterile circumstances. It was shown that ultrasound can help localize nerve lesions intraoperative and guide the surgical approach in peripheral neurosurgery [8]. In our opinion intraoperative ultrasound has the potential to guide the drill to avoid radial nerve lesions in the spiral groove in open reduction and internal fixation of humeral fractures.

A limitation of this study is the small sample size. A larger clinical study population should confirm our results.

## Conclusion

With this study, we show that ultrasound can reliable visualize the screw tips and its relation to the radial nerve. In case of neuropathic pain and/or nerve lesion presenting after open reduction and internal fixation of a humeral fracture, ultrasound is a promising diagnostic tool to evaluate the site of operation for iatrogenic or trauma related nerve pathologies. The probability of nerve damage or nerve irritation due to the drilling or due to the implanted material (cortical overlapping screw tips) can be adequately assessed by ultrasound.

Furthermore, ultrasound can be used as additional tool to guide drilling in open reduction and internal fixation of humeral fracture. The intraoperative ultrasound visualizing of the radial nerve and ultrasound guidance of the drill in zones of risk (drill in direction of the spiral groove) might be a possibility to prevent radial nerve damage by open reduction and internal fixation of humeral fractures. Whether secondary radial nerve damage can really be prevented or not by using intraoperative ultrasound visualization of the nerve and to guide the direction of drilling has to be subject of further studies.
